# Structural rearrangements as a recurrent pathogenic mechanism for *SETBP1* haploinsufficiency

**DOI:** 10.1186/s40246-024-00600-0

**Published:** 2024-03-22

**Authors:** V. Alesi, S. Genovese, M. C. Roberti, E. Sallicandro, S. Di Tommaso, S. Loddo, V. Orlando, D. Pompili, C. Calacci, V. Mei, E. Pisaneschi, M. V. Faggiano, A. Morgia, C. Mammì, G. Astrea, R. Battini, M. Priolo, M. L. Dentici, R. Milone, A. Novelli

**Affiliations:** 1 Laboratory of Medical Genetics, Translational Cytogenomics Research Unit, Bambino Gesù Children Hospital, IRCCS, 00146 Rome, Italy; 2 Operative Unit of Medical Genetics, Great Metropolitan Hospital of Reggio Calabria, 89100 Reggio Calabria, Italy; 3 Department of Developmental Neuroscience, IRCCS Fondazione Stella Maris, 56125 Pisa, Italy; 4 Department of Clinical and Experimental Medicine, University of Pisa, 56100 Pisa, Italy; 5 Medical Genetics Unit, Bambino Gesù Children Hospital, IRCCS, 00146 Rome, Italy

**Keywords:** *SETBP1*, Optical genome mapping, OGM, Complex rearrangement, Translocation, RASopathy

## Abstract

Chromosomal structural rearrangements consist of anomalies in genomic architecture that may or may not be associated with genetic material gain and loss. Evaluating the precise breakpoint is crucial from a diagnostic point of view, highlighting possible gene disruption and addressing to appropriate genotype–phenotype association. Structural rearrangements can either occur randomly within the genome or present with a recurrence, mainly due to peculiar genomic features of the surrounding regions. We report about three non-related individuals, harboring chromosomal structural rearrangements interrupting *SETBP1*, leading to gene haploinsufficiency. Two out of them resulted negative to Chromosomal Microarray Analysis (CMA), being the rearrangement balanced at a microarray resolution. The third one, presenting with a complex three-chromosome rearrangement, had been previously diagnosed with *SETBP1* haploinsufficiency due to a partial gene deletion at one of the chromosomal breakpoints. We thoroughly characterized the rearrangements by means of Optical Genome Mapping (OGM) and Whole Genome Sequencing (WGS), providing details about the involved sequences and the underlying mechanisms. We propose structural variants as a recurrent event in *SETBP1* haploinsufficiency, which may be overlooked by laboratory routine genomic analyses (CMA and Whole Exome Sequencing) or only partially determined when associated with genomic losses at breakpoints. We finally introduce a possible role of *SETBP1* in a Noonan-like phenotype.

## Background

Chromosomal structural rearrangements (CSR) have been known for a long time as a potential etiological mechanism in human diseases [1–4]. The most common ones are reciprocal translocations, consisting of the mutual exchange of non-homologous genomic material between two chromosomes, and accounting for approximately 0.1–0.2% of human population [5]. However, their prevalence is likely underestimated, only referring to karyotype-detectable ones. Structural rearrangements arising from more than two chromosomal breaks are usually considered as complex rearrangements.

Although CSR do not usually result in a clinical phenotype and can segregate across generations, an increased risk of congenital anomalies is documented, which is two or three times higher than in the general population [6]. Different mechanisms can be associated with a pathological outcome, such as loss of genomic material at the breakpoints, genes physical disruption, positional effects, chimeric gene formation, and alteration of genomic environment having a role in gene regulation. Therefore, CSR detection, together with a fine characterization of the breakpoints, is crucial from a diagnostic point of view.

Despite their acknowledged importance, structural rearrangements are often overlooked during routine genetic analyses, due to the technical limitations of the currently used genomic platforms, mainly represented by Chromosomal Microarray Analysis (CMA) and Exome Sequencing. In fact, while these technologies are exponentially increasing the diagnostic detection rate, revealing copy number and sequence variants all over the genome, they cannot provide clear information about structural variants. Karyotype is still considered the only technique able to detect CSRs in laboratory routine. However, the resolution level of optical microscopy does not allow a fine characterization of the breakpoints, limiting the diagnostic application and utility of cytogenetic analyses.

The recent introduction and availability of genomic techniques such as Whole Genome Sequencing (WGS) and Optical Genome Mapping (OGM) is enabling a rapid detection and a fine characterization of chromosome breakpoints at a high resolution, providing further information on molecular mechanisms underlying genetic diseases.

Here, we describe three non-related additional patients presenting with different types of CSR interrupting the SET Binding Protein 1 (*SETBP1*) gene.

*SETBP1* haploinsufficiency disorder (*SETBP1*-HD) (MIM#616,078) is caused by either heterozygous gene deletions or loss-of-function (LoF) variants. It is associated with a genetic condition, mainly characterized by intellectual disability (ID), also known as *“Intellectual disability, autosomal dominant 29”* (MRD29). Expressive speech and language impairment appear as a prominent feature of the disorder in association with behavioral problems and mild dysmorphisms, especially in patients with microscopic and submicroscopic 18q12 deletions involving *SETBP1* [7–9]. Pathogenic *SETBP1* gain of function variants, on the other hand, are associated with an ominous different syndromic condition (i.e. Schinzel-Giedion syndrome), characterized by severe intellectual disability, distinctive facial features, and multiple congenital malformations including skeletal abnormalities, genitourinary and renal malformations. It mainly differs from *SETBP1* haploinsufficiency by neurodegeneration and by the lack of a preeminent neurobehavioral component [10].

High-resolution CMA and genomic sequencing are the methods utilized so far to diagnose *SETBP1* microdeletion and variants [10].

In our patients molecular diagnosis was obtained through a combined approach based on Optical Genome Mapping and WGS, providing a fine characterization of the genomic sequence at the breakpoints, and suggesting CSR as a new etiological mechanism for *SETBP1*-HD.

### Case report

Subject ID1: Subject ID1 is a 10-year-old boy and he was born at 37th gestational week by emergency cesarean section due to fetal sufferance. Family history was positive for generalized epilepsy due to neonatal hypoxia and for Hodgkin lymphoma in the maternal line. Due to a previous pregnancy interrupted at the 21st gestational week (therapeutic abortion) for transposition of the great vessels in a male fetus, an amniocentesis was performed, detecting a de novo balanced translocation between the long arms of chromosomes 15 and 18 (karyotype 46 XY,t(15;18)(q24;q21).

Birth weight was 2,680 g (22th centile, 0.77 SD), length was 46 cm (10th centile, -1.3 SD), and occipito-frontal circumference (OFC) was 35 cm (85th centile, 1.02 SD). Apgar scores were 6 and 8 at the minutes 1and 5, respectively. Neonatal jaundice was treated with phototherapy. Transient-evoked otoacoustic emissions resulted pass on the right side and refer on the left one. Auditory brainstem responses were normal bilaterally. Transfontanellar ultrasound detected ventricular enlargement and right choroid plexus cyst. Bilateral cryptorchidism was detected and corrected by orchidopexy at the age of two, while abdominal ultrasound was normal.

He was breast-fed from the 5th day-life after initial suction difficulties. Weaning was characterized by chewing difficulties and reduced tolerance for different tastes and textures. Motor development was delayed and characterized by relevant hypotonia: he obtained head control at 5 months and autonomous ambulation at the age of 19 months. He presented with consistent speech delay; expressive language was far more impaired than receptive abilities, and the child was able to utter vowels, syllables, onomatopoeia but not words, with a reduced phonetic inventory. Speech articulation was evidently hard, and he obtained a diagnosis of developmental verbal dyspraxia.

Social interaction and non-verbal communicative intent were valid and mediated by gestures. Play schemes were poorly organized. Oppositional-defiant behavior, brief attention and prestation discontinuity characterized his functioning. Joint laxity and poor motor coordination were evident.

At the age of 4 an episode of febrile convulsion occurred. Awake electroencephalogram showed bilateral posterior slow rhythmic activity. Brain MRI detected mild gyral asymmetry on anterior temporal convolutions, not considered as a pathologic finding. Ophthalmological evaluation showed hypermetropia. Echocardiography detected patent ductus arteriosus with left-to-right shunt.

At 4 years he was tested for his cognitive development using Griffiths Mental Development Scales-II edition, and moderate developmental delay (DD) was diagnosed (Developmental Quotient below 3 standard deviations). At the age of 7, Leiter-3 (non verbal intelligence quotient 54) and Vineland Adaptive Behavior Scale-II edition (worse communicative and motor abilities in comparison with better social and day life abilities) were administered and confirmed moderate ID.

Conners’ Parents Rating Scales were positive for Attention Deficit Hyperactivity Disorder (ADHD), and clinical evaluation confirmed this condition.

**Fig. 1:**
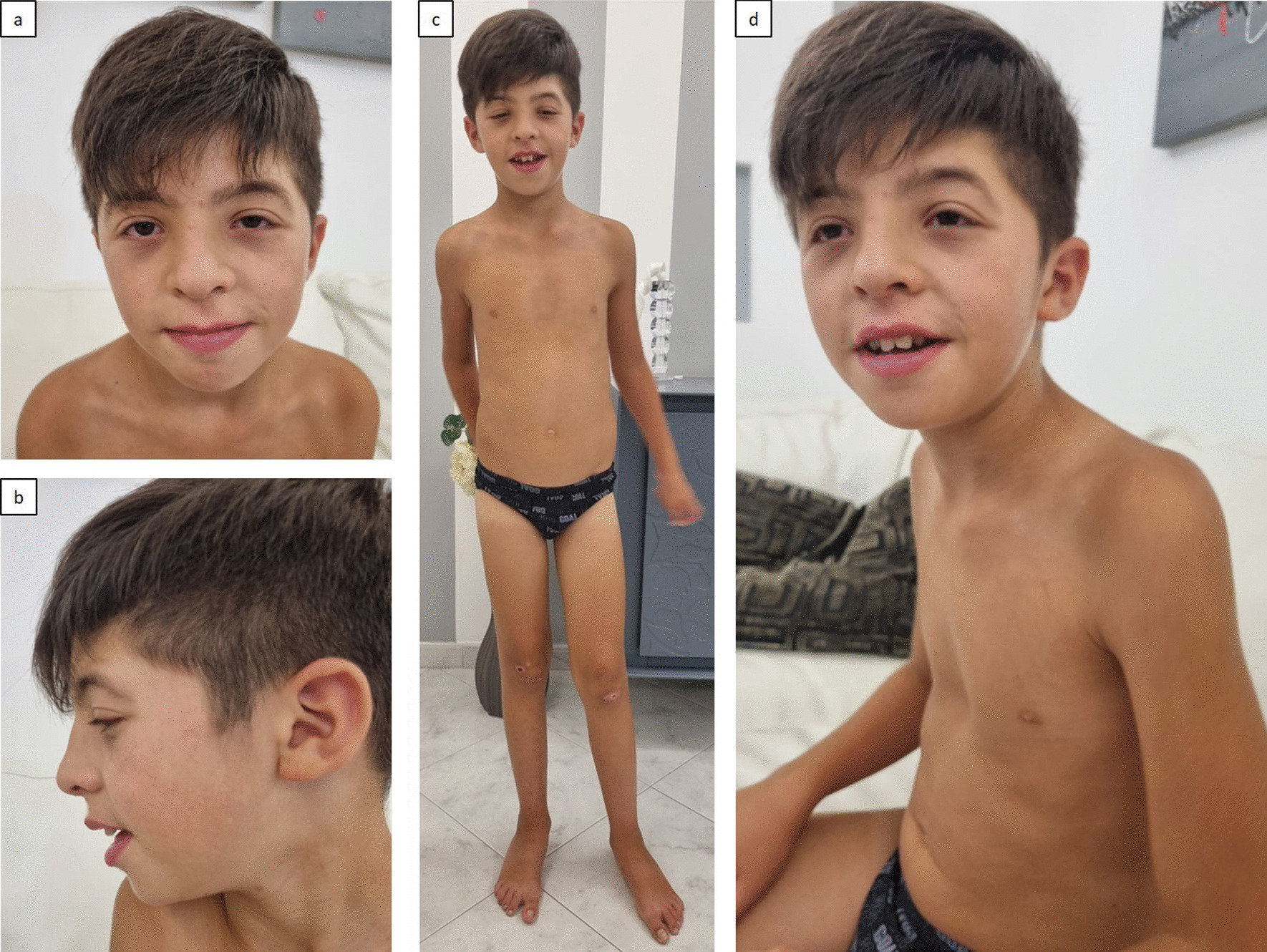
Patient ID 1 physical features. **a** bushy arched eyebrows, wide spaced eyes, bilateral epichantus, ptosis (prevalent in left eye), downslanting palpebral fissures, enlarged nasal sella with pressed columella and arched nasal pinnae, marked nasal philtrum, pointed chin; **b** chubby ear-lobes, low set-posteriorly rotated ears; **c** short neck, wide-spaced nipples, pes planus; **d** tented upper lip, pectus excavatum, widely spaced nipples

Physical examination revealed arched eyebrows, hypertelorism, bilateral epicanthus, ptosis, down-slanting palpebral fissures, enlarged nasal sella with pressed columella and arched nasal pinnae, marked nasal philtrum, tented upper lip, retro-rotated low-set ears with chubby ear-lobes, pointed chin, short neck, pectus excavatum, wide-spaced nipples, pes planus (Fig. 1). OCF had grown within the upper limits of the norm. Globally, his phenotype resembled a Noonan syndrome (NS) -like condition.

Muscular hypotonia, evident in the first years of life, led to *DMPK* gene analysis in order to exclude myotonic dystrophy, which resulted normal (12 CTG triplets).

Loss of genetic material in the translocation regions had been excluded by array Comparative Genomic Hybridization (a-CGH), although it revealed a de novo 276 kb deletion on the short arm of chromosome X (arr[GRCh38] Xp22.1(22,818,207_23,093,865) × 0) involving *DDX53*.

A Noonan-customized gene panel.

Exome sequencing by means of Twist Human Core Exome Kit (Twist Bioscience), revealed heterozygous mutations in genes related to recessive conditions, therefore they were not considered causative of our patient’s phenotype (c.661G > A (p.Ala221Thr) inherited from his father in *TMCO1*; 2791A > T (p.Asn931Tyr) in *SPTBN4* and c.8713C > T (p.Arg2905Cys) in *ANK3* inherited from his mother).

Subject ID2: Subject ID2 is a 4-year-old male, the only child of non-consanguineous parents. Family history was unremarkable. Pregnancy was uneventful. Spontaneous delivery was at 41 + 2 weeks. Birth weight was 3,420gr (39th centile, 0.29 SD), length 53 cm (91th centile, 1.32 SD), and OFC 35 cm (53th centile, 0 SD). A cerebral ultrasound performed after birth was normal. At 6 months of age urinary ultrasound documented vescico-uretheral reflux and mild right caliectasis of 5 mm. Motor development was delayed: he walked unsupported at 15 months. At 2 years speech delay was noticed. The patient has a discrete verbal comprehension but still no verbal capacity. Vocalization was absent and he only started producing afinalistic babbling sounds at the age of 4 and reported the word “mommy”. He required assistance in hygiene and dressing. Griffith cognitive scale performed at 45 months displayed a mild DD with severe language impairment; Developmental Quotient was 71, with disharmonious profile between the various index (Locomotor abilities 84, Personal and Social abilities 75, Hearing and Language abilities 24, Eye and Hand Coordination 84, Performance abilities 84, Practical Reasoning 75). At 3 years and 1 month, he started speech therapy and he was attending primary school with support.

Audiometric test was normal. Growth parameters were within normal range. He did not present gastrointestinal problems.

At the last physical evaluation (3 years and 10 months), growth parameters were: weight 17 kg (70th centile, 0,51 SD), stature 107 cm (93th centile, 1.5 SD), and OFC 50,6 cm (58th centile, 0.2 SD). Physical examination disclosed dysmorphic facial features and signs that remained unchanged over the time, including triangular face, hypertelorism, epicanthus, narrow palpebral fissures, prominent nasolabial fold, normal setting of the ears, high narrow palate, clinodactyly of the fifth toe.

Genetic analyses, including FRAXA, 15q MS-MLPA for Angelman syndrome exclusion and array-CGH at an average resolution of 100 kb, tested negative. Karyotype analyses revealed a de novo reciprocal translocation involving the long arm of a chromosome 18 and the short arm of a chromosome 16, reported as 46,XY,t(16;18)(p13.2;q21.1).

Subject ID3: Subject ID3 is an 8 year and 6 months-old boy with absent speech, DD, and facial dysmorphisms slightly resembling a RASopathy. He was born at 40 weeks of gestation by cesarean section for dystocic presentation after a pregnancy obtained with heterologous artificial reproductive technique (sperm donor) and complicated by threatened abortion occurring during the first months and maternal hyperglycemia treated with diet. Birth weight was 3250 kg (30th centile, -0.5 SD), length was 51 cm (60th centile, + 0.2 SD), and OFC was 36 cm (87th centile, + 1.1 SD). Apgar scores were 9 and 9 at minutes 1 and 5, respectively. Soon after birth, he was admitted to Neonatal intensive care unit for respiratory distress, right subtotal pneumothorax, partial left pneumothorax, treated with pleural drainage and requiring directional positive air-way pressure assistance. At birth, bilateral ptosis with severe involvement of the left eye was noticed. Weaning was characterized by chewing difficulties and reduced tolerance for different tastes and textures. He presented with DD with generalized hypotonia (first words at 4 years but lost soon afterwards, walked unassisted at 3 years with a wide base gait, no sphincter control at 8 years). At nine months, he experienced repeated febrile seizures treated with Phenobarbital and Sodium Valproate until the age of 6. He is currently without seizures. At our first examination (8 months) growth parameters were: weight 7.8 kg (13th centile, -1.13 SD), height 74 cm (91th centile, + 1.34 SD), and OFC 45 cm (57th centile, + 1.18 SD). An abdominal ultrasound exam revealed mild left pielic ectasia. Bilateral cryptorchidism was also detected.

Dysmorphic facial features included high anterior hairline, high forehead with mild bitemporal narrowing, bushy eyebrows, wide and high nasal bridge, mild widely spaced eyes, epicanthic folds, bilateral ptosis with blepharophimosis, low-set and large, fleshy ears, short nose with anteverted nostrils, deep nasolabial folds, broad long philtrum, short tongue frenulum surgically treated at 9 months, narrow palate, long pointed chin. He presented with a happy demeanor.

Palpebral ptosis was surgically treated at 3 and 7 years, with partial resolution.

A brain MRI did not reveal structural brain anomalies, echocardiogram and audiologic evaluations were normal.

Clinical evaluation at 8 years confirmed DD and absent speech for which he required speech therapy and the introduction of augmentative and alternative communication (AAC). He was also diagnosed with ADHD with autistiform traits characterized by repetitive movements and high anxiety levels. He attends primary school with support. At the last physical examination at the age of 8 years and 6 months, growth parameters were: weight 23 kg (12th centile, -1.18 SD), height 133 cm (69th centile, + 0.59 SD), and OFC 52.5 cm (54th centile, 0 SD). Dysmorphic features were confirmed, mild pectus excavatum with wide-paced nipples, pes planus, and mild constipation were recorded. He continues on presenting with an unusual happy demeanor with unprovoked laughing.

CMA analysis detected 5 microdeletions, involving chromosomes 2, 6, and 18:

- arr[GRCh38] 2q37.1(230,834,026_230,861,014) × 1,2q37.3(237,414,009_238,552,715) × 1,6q27(164,870,831_165,833,376) × 1,6q27(166,036,017_166,385,898) × 1,18q12.3q21.1(44,767,804_48,427,129) × 1
- The 18q12.3q21.1 microdeletion extends for 3.65 Mb and involves 16 OMIM genes, including *SETBP1*, which mapped at the proximal breakpoint of the region, resulting partially deleted.

## Results

OGM and WGS were performed to refine the breakpoints of the structural rearrangements.

**Fig. 2:**
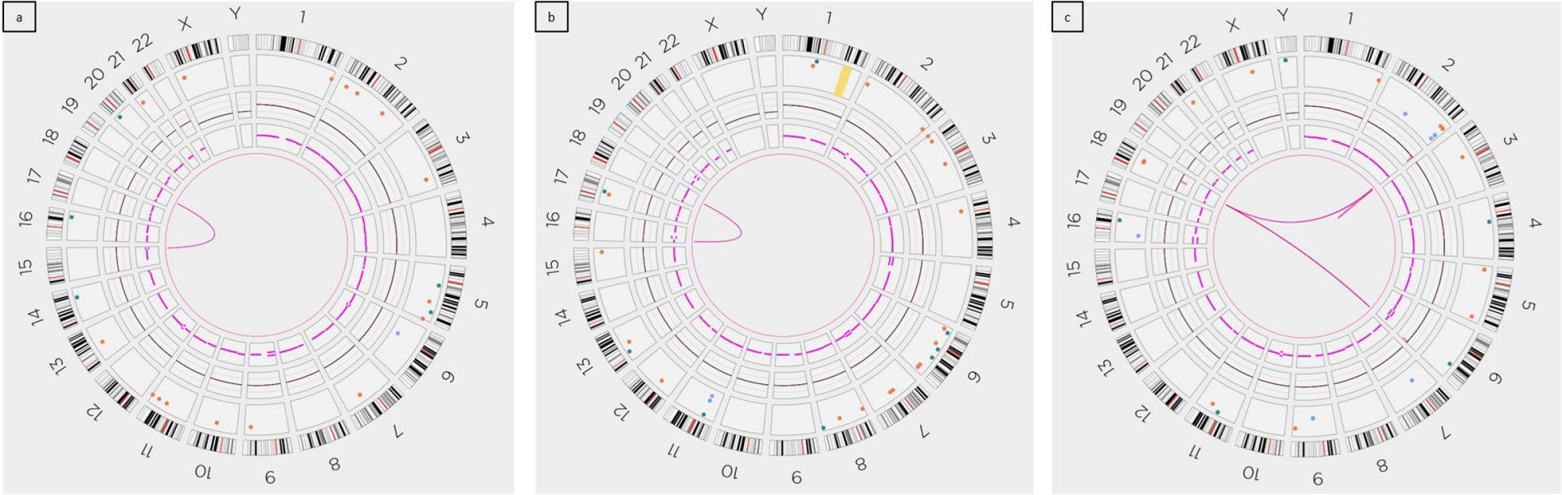
Circle plots: Optical Genome Mapping shows: **a** a reciprocal translocation between chromosomes 15 and 18 in patient ID1; **b** a reciprocal translocation between chromosomes 16 and 18 in patient ID2; **c** a complex rearrangement between chromosomes 2, 6, and 18 in patient ID3

**Fig. 3:**
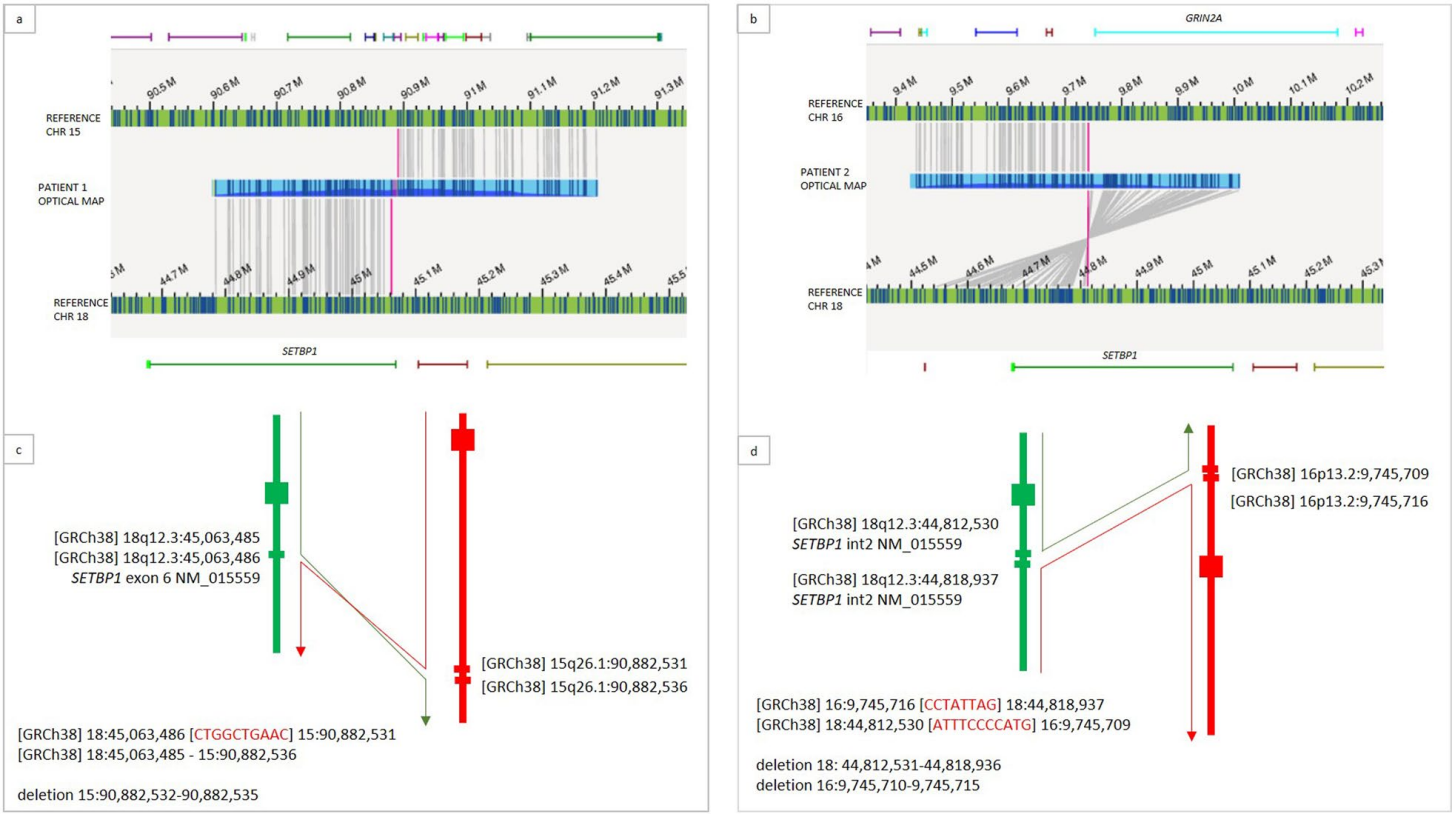
Breakpoint characterization on patient ID1 e ID2: OGM characterized the balanced translocation as: **a** t(15;18)(q26.1;q12.3) in patient ID1; **b** t(16;18)(p13.2;q12.3) in patient ID2. The patients’ optical map overlapping the breakpoint (blue segment) is paired with chromosome references (green segments). Matched labels between patient’s and reference maps are reported as grey lines connecting them. The breakpoints have been finely defined by WGS, showing sequence alterations as a result of DNA repair mechanisms: few bases deletions and insertions at breakpoints. The two resulting derivative chromosomes have been consequently reconstructed as shown schematically (**c**, **d**)

**Fig. 4:**
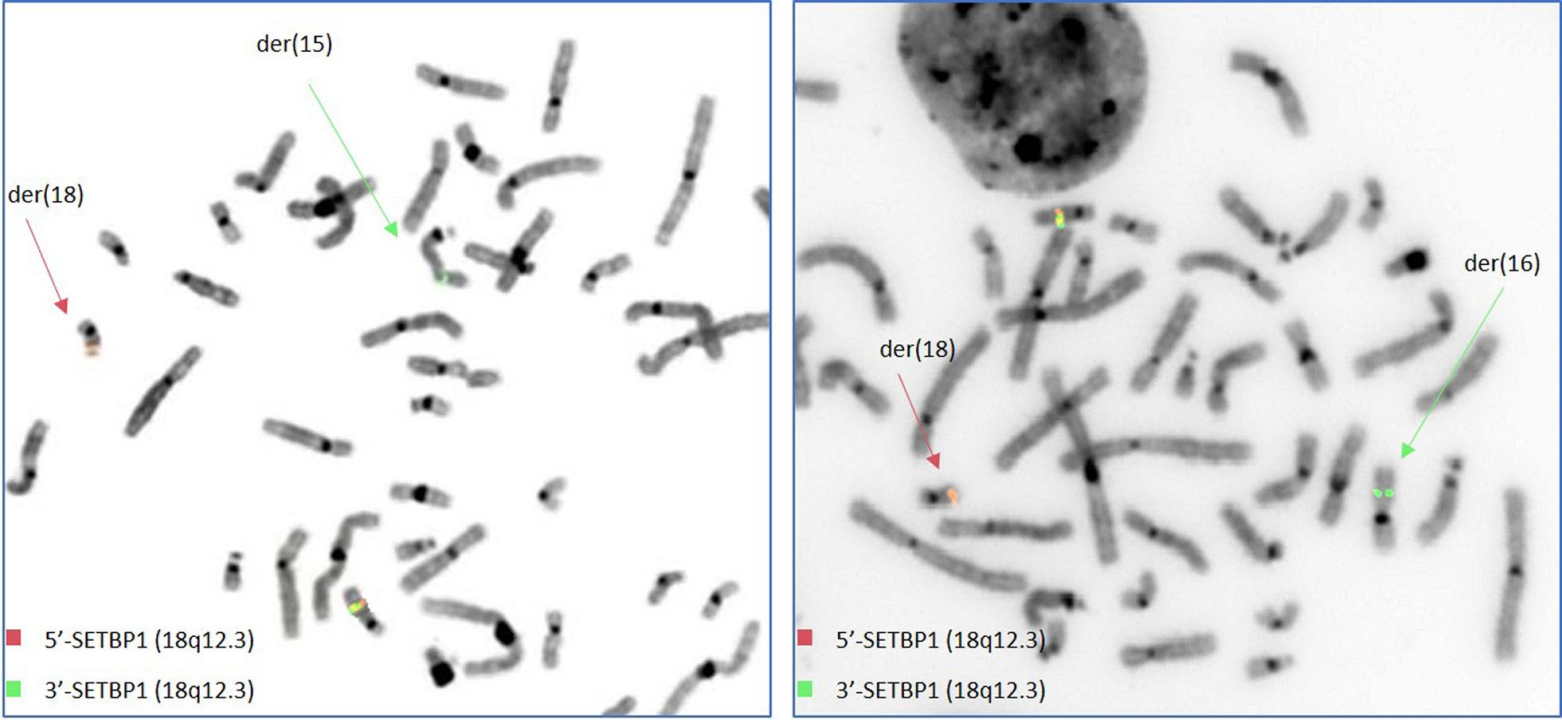
FISH analysis by means of locus specific oligonucleotide probes showing *SETPB1* break in patient ID1 (**a**) and ID2 (**b**)

Subject ID1: the presence of the known reciprocal translocation was confirmed and better characterized as t(15;18)(q26.1;q12.3) (Fig. 2a). The breakpoints on the two involved chromosomes were mapped at [GRCh38] 15:90,882,532 and 18:45,063,485 respectively (Fig. 3a, c). A 4 bp deletion was present at 15q26.1 breakpoint (within exon 16 of *FURIN*, downstream the stop codon*)*, while a 10 bp insertion of unknown origin material was detected at the breakpoint level on derivative 18. The breakpoint on chromosome 18 occurred within exon 6 of *SETBP1* [NM_015559] (Fig. 4a). The breakpoints were consequently defined according to ISCN as:

- NC_000015.10:g.90,882,532_qterdelins[CAAGTCGGTC;NC_000018.10:g.45,063,486_qter]
- NC_000018.10:g. 45,063,485_qterdelins[NC_000015.10:g.90,882,536_qter]

Subject ID2: OGM and WGS allowed characterizing the reciprocal translocation as t(16;18)(p13.2;q12.3) (Fig. 2b), having breakpoints at [GRCh38] 16:9,745,716 and 18:44,812,531 (Fig. 3b, d). WGS revealed a 6 bp deletion at the breakpoints on chromosome 16, in a gene desert region. The breakpoint on chromosome 18 occurred within intron 2 of *SETBP1*, where the presence of a 6.4 kb deletion was also evidenced. Two small insertions were detected on the two derivative chromosomes, where the breakpoints occurred, spanning for 8 bp and 11 bp respectively. The breakpoints wwere consequently defined according to ISCN as:

- NC_000016.10:g.pter_ 9,745,716delins[CCTATTAG;NC000018.10:g.44,818,937_qterinv]
- NC_000018.10:g.44,812,531_qterdelins[ATTTCCCCATG;NC_000016.10:g.16:9,745,709_pterinv]

FISH analysis, using oligonucleotide locus-specific custom probes, was performed in subjects ID1 and ID2, confirming the interruption of *SETBP1* and showing, in both cases, the presence of the *SETPB1*-3’ signal in ectopic position, on the translocation partner chromosome (Fig. 4).

### Subject ID3

OGM confirmed the five deletions previously detected by array-CGH on chromosomes 2, 6, and 18, and showed a physical association of the involved chromosomes in a complex genomic rearrangement (Fig. 2c).

**Fig. 5:**
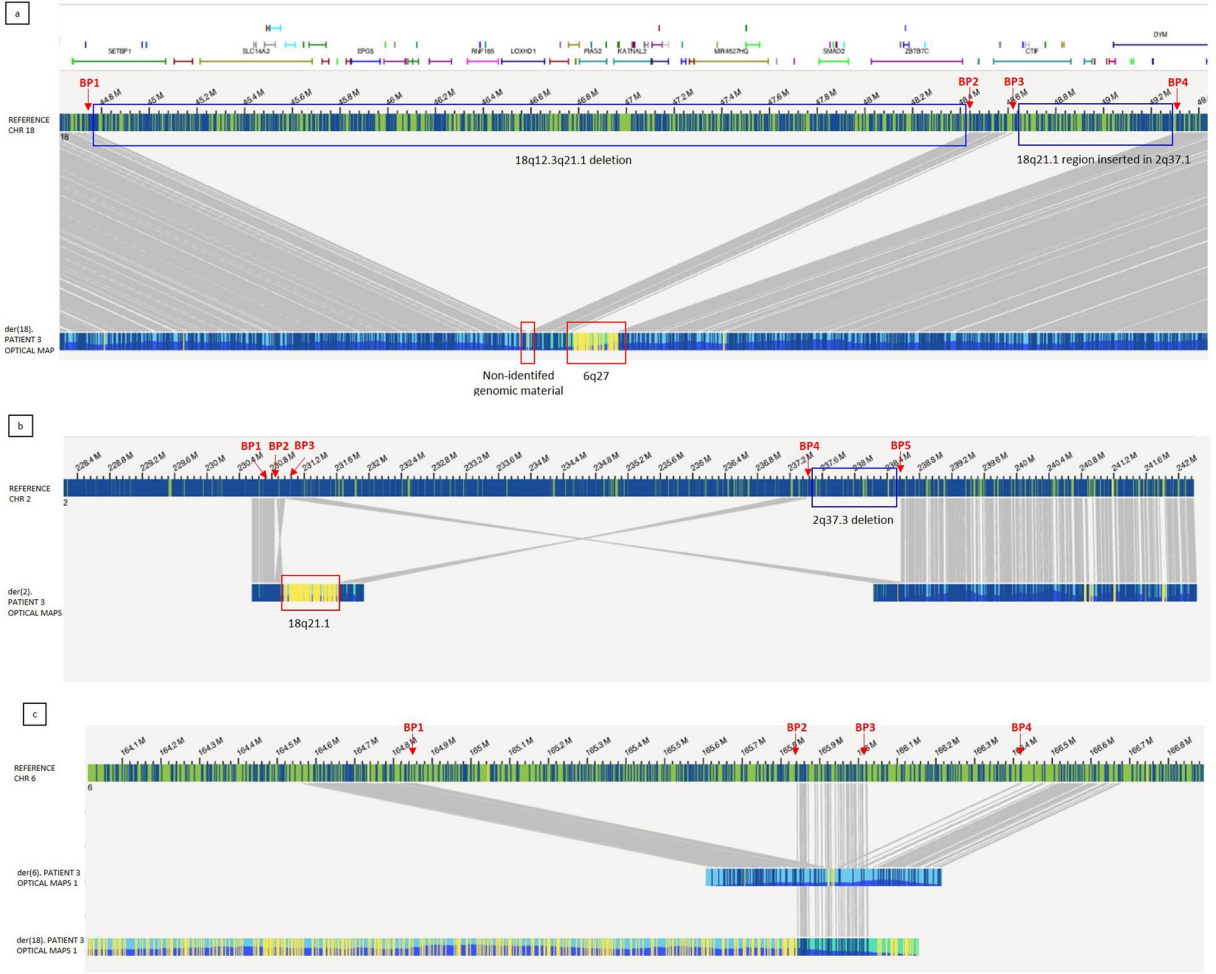
Optical Map of patient ID 3: Patient’s optical map (bottom) is compared to a reference map (up); matched labels between patient’s and reference maps are reported as grey lines connecting them. **a** der(18): Four breakpoints have been detected on chromosome 18 (red arrows), resulting in a complex rearrangement: A 13 kb of non-identified material inserts at 18q12.3, BP1 level (yellow unmatched labels in the first in red box), followed by a 173.8 kb portion from 18q21.1 (BP2-BP3 region). A 180 kb region from 6q27 inserts with opposite orientation (yellow unmatched labels in the second red box) and rejoins with chromosome 18 at BP4 level. BP1-BP2 (3.65 Mb) genomic portion results to be deleted (first blue box), while BP3-BP4 portion was inserted in chromosome 2, at 2q37.1 (second blue box). SETBP1 is represented as a green bar on the top of the image, interrupted by the BP1-BP2 deletion. **b** der(2): Five breakpoints have been detected on chromosome 2 (red arrows), resulting in a complex rearrangement: BP2-BP3 region inverts on itself, and associates with BP1, resulting in the BP1-BP2 deletion (27 kb). 18q21.1 region inserts in inverted orientation (yellow unmatched labels in the red box), followed by BP4-BP3 region also inserted in opposite orientation (2q37.3q37.1). BP3 physically associates with BP5, resulting in BP4-BP5 deletion (1.1 Mb, blue box). **c** der(2): Five breakpoints have been detected on chromosome 2 (red arrows), resulting in a complex rearrangement: BP2-BP3 region inverts on itself, and associates with BP1, resulting in the BP1-BP2 deletion (27 kb). 18q21.1 region inserts in inverted orientation (yellow unmatched labels in the red box), followed by BP4-BP3 region also inserted in opposite orientation (2q37.3q37.1). BP3 physically associates with BP5, resulting in BP4-BP5 deletion (1.1 Mb, blue box).

In particular: five different breakpoints (BP) were observed on chromosome 2, four on chromosome 6, and four on chromosome 18. The reconstruction of the physical maps allowed determining the structure of the three resulting derivative chromosomes (Fig. 5):

- _der(18) (Fig. 5a): The first breakage (BP1) occurred at 18q12.3, at intron 2 level of *SETBP1* gene ([GRCh38] 18:44,759,272)], where a short unknown genomic portion, about 13 kb in size, was inserted. A second BP occurred at 18q21.1 (BP2), resulting in the loss of the proximal region 18q12.3q21.1 (BP1-BP2), as previously shown by array-CGH. Therefore, the 13 kb unknown material resulted to be directly ligated with 18q21.1 region. A third BP (BP3) occurred about 173.8 kb downstream, where a 179.9 kb region from chromosome 6 (6q27) was inserted in an inverted orientation. This latter joins to BP4, at 18q21.1, about 692.9 kb apart from BP3. The excluded BP3-BP4 region is inserted in der(2), at 2q37.1.
- _ der(2) (Fig. 5b): Two BPs (BP1 and BP2) occurred at 2q37.1 and the genomic region between them was lost, resulting in a 27 kb deletion. The third breakpoint about 96 kb downstream from BP2 and the BP2-BP3 region inverted on itself, followed by the 18q21.1 insertion. A forth BP (BP4) occurred at 2q37.3, and BP4-BP3 region ligated downstream the 18q21.1 insertion, in inverted orientation. BP4-BP5 region was lost, resulting in a 1.1 Mb deletion.
- _ der(6) (Fig. 5c). The first BP (BP1) occurred at 6q27, where 15 kb of non-identified genomic material is inserted. The second and the third breakpoints (BP2 and BP3) occurred respectively 1.2 Mb and 1.4 Mb from BP1. The BP1-BP2 region (963 kb) was lost while BP2-BP3 region inserted within the long arm of der(18). A further deletion occurred between BP3 and BP4 (350 kb).

**Fig. 6:**
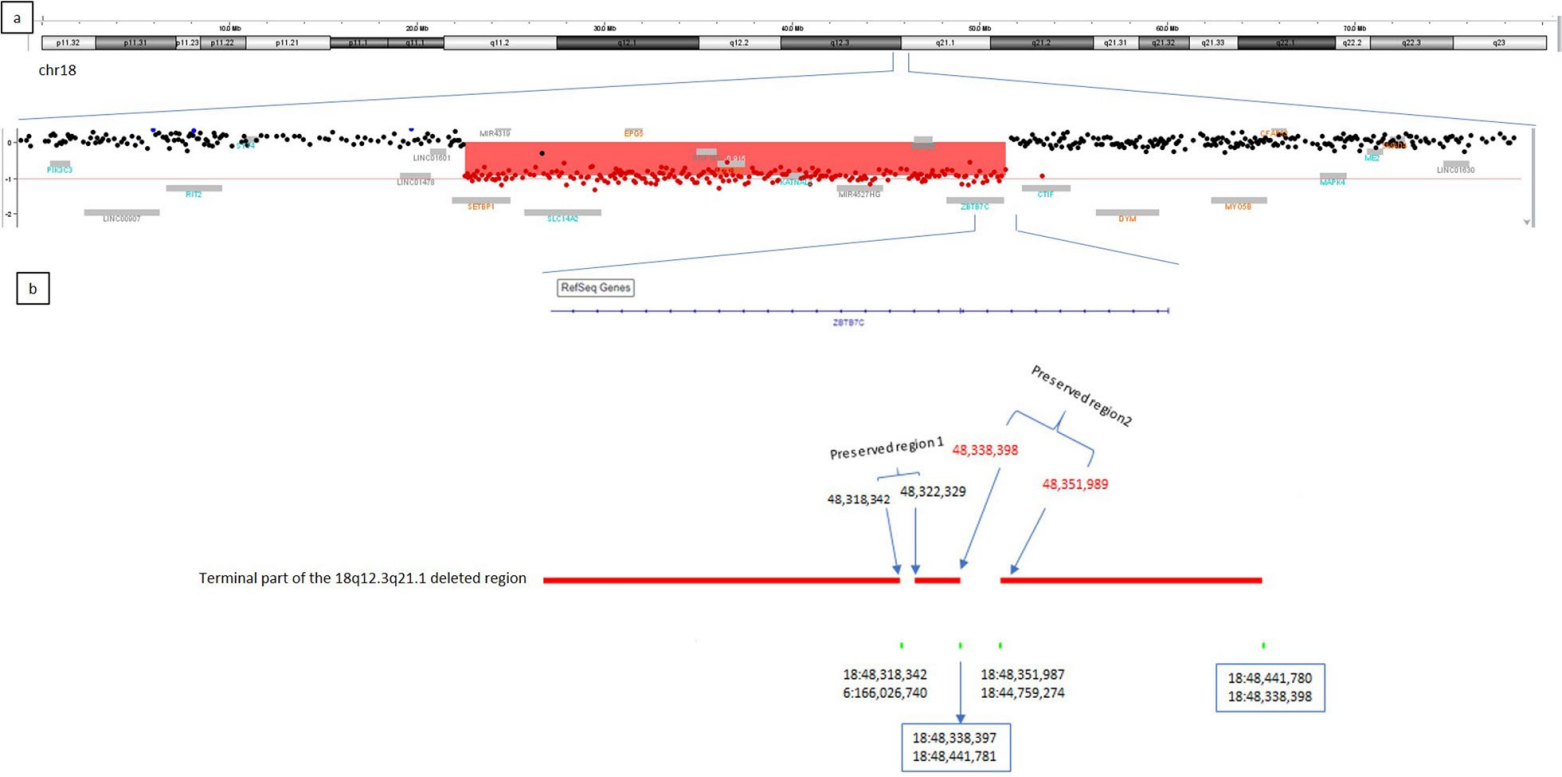
Characterization of the 18q12.3q21.1 microdeletion: **a** array-CGH image showing the deleted region as a downward shift of oligonucleotide probes (red highlighted). The Deletion breakpoints are mapped within between SETBP1 (left) and downstream ZBTB7C. **b** Enlargement of the microdeletion terminal portion: WGS detecting four further breakpoints and the preservation of two small regions, identified as 1 and 2. Region 1 results to involved in a rearrangement with der(6), while region 2, orientation inverted, represents the unknown genomic material previously detected by OGM. Genomic positions are reported according to GRCh38 assembly

WGS, used to characterize the *SETBP1* breakpoint at a nucleotide level, revealed an even more complex structural rearrangement than previously supposed, highlighting several further genomic ruptures and fusions. In particular, four new breakpoints have been detected at 18q21.1 (Fig. 6b). Consequently, the 18q12.3q21.1 region was shown to be not entirely deleted, as previously supposed, but composed of three different deletions. The preserved two regions were 3.9 kb and 13.6 kb in size; the latter, with inverted orientation, was recognized as the unknown material shown on der(18) by means of OGM.

The *SETBP1*breakpoint was therefore defined as:

- NC_000018.10:g.44,759,272_44,759,273ins[NC000018.10:g. 48,338,398_48,351,989inv]

## Discussion

We report on 3 individuals, harboring different structural rearrangements which interrupt the coding sequence of *SETBP1* and likely lead to gene haploinsufficiency. *SETBP1*-HD is either caused by heterozygous *SETBP1*deletions or LoF variants. *SETBP1*-HD has been reported in 48 individuals so far [10–13].

The main clinical features of the condition have been well outlined and include mild motor developmental delay, severe speech impairment, a wide range of intellectual disability ranging from mild to severe (although with a quarter of cases with normal to borderline intelligence quotient), hypotonia in childhood, vision impairment, and behavior problems mainly characterized by attention deficits (59% of reported individuals), and hyperactivity (35%) [10, 13] (Table 1). Typically, children with *SETBP1-*HD present with speech delay (first words by 18 months in 50%) due to a severe childhood apraxia of speech (CAS) (observed in 80%) [6, 10, 14–17]. Individuals with *SETBP1*-HD may also present with a mild-to-moderate receptive language disorder. However, in about one third of subjects, receptive language may be even better than expressive language [17].

**Table 1:** Main clinical features in individuals with *SETBP1*-HD (modified from Jansen et al. 2021)

Clinical features	*n* = 34 from Jansen et al. 2021	Subject ID1	Subject ID2	Subject ID3
Gender (male:female)	19:15 (56% male)	Male	Male	Male
Motor developmental delay	97%	+	+	+
Speech delay	97%	+	+	+
Intellectual Disability/Developmental Delay	77%	+ (Mod)	+ (Mild)	+
Facial Dysmorphisms	+	+	+	+
Ptosis	+	+	+	+
Short palpebral fissures	+	–	+	+
Epicanthal folds	na	+	+	+
Broad nasal bridge	+	+	+	+
Wide spaced eyes	+	+	+	+
Short nose with anteverted nostrils	na	+	+	+
Deep nasolabial folds	na	–	+	+
High narrow palate	+	–	+	+
Vision impairment	48%	+ (HM)	–	–
Hearing impairment	9%	–	–	–
Hypotonia	52%	+	+	+
Seizures	21%	+	–	+
Febrile	15%	+	–	+
Ankyloglossia/short frenulus	23% (25% in Morgan et al. 2021)	–	–	+
Undescended testicles (males)	14%	+	–	+
Behavior problems	76%	+	–	+
Anxieties	24%	–	–	+
Hyperactivity	35%	+	+	+
Attention/concentration deficit	59%	+	–	+
Diagnosed ADHD	18%	+	–	+
Temper tantrums	24%	–	–	–
Aggressive behavior	21%	–	–	–
Sleep problems	12%	–	–	–
Self-mutilation	8%	–	–	–

HM: Hypermetropia; MOD: Moderate; na: not assessed

The most common behavior problems are attention/concentration deficits, hyperactivity and impulsivity, leading in many instances to a diagnosis of ADHD. Other problems include anxiety, autism spectrum disorder (ASD), sleep disturbances, self-injury, and other aggressive behaviors [13]. Previous authors did not focus on a distinctive facial gestalt, although the presence of a mild overlap in certain facial dysmorphisms which included ptosis, blepharophimosis, a broad nasal bridge, hypertelorism, a full nasal tip, and a high arched palate was evidenced. Less frequently reported features are ankyloglossia (25%), febrile seizures (20%), and genital anomalies, such as cryptorchidism (20%) [13].

Systematically assessing the clinical and facial features for the three individuals with pathogenic LoF rearrangements of *SETBP1*, summarized in Table 1**,** we found an overlapping of their clinical presentations with those described in the literature, including less frequently reported features such as febrile seizures (subjects 1 and 3), ankyloglossia (subject ID3), and cryptorchidism (subjects ID1 and ID3).

By reviewing the facial features, we found the occurrence of the main facial features commonly observed in *SETBP1*-HD presentation, represented by ptosis, blepharophimosis, a broad nasal bridge, and wide spaced eyes. We also evidenced epicanthic folds, a short nose with anteverted nostrils and deep nasolabial folds (Table 1). These signs were also reviewed on available clinical data and pictures of previously reported individuals [10, 13] and they were found to be consistent.

Two individuals presented with facial and clinical features reminiscent of a RASopathy. Some of the most characteristic facial features resembling Noonan Syndrome (ptosis, broad nasal bridge, hypertelorism) have also previously described as common dysmorphisms in *SETBP1*-HD [10, 13] and, together with other signs (i.e.: pectus excavatum with wide-spaced nipples) may evoke this clinical suspicion. However, differently from RASopathies, *SETBP1*-HD typically presents with short instead of long palpebral fissures, and with normal stature. Furthermore, cardiopathy is not frequent in *SETBP1*-HD.

Intriguingly, *SETBP1* interacts with RAS/MAPK cascade by inhibiting the activity of the tumor suppressor PROTEIN PHOSPHATASE 2A (PP2A), which regulates this pathway through the stabilization of SET, another important tumor suppressor. Although until recently these mechanisms have been chiefly studied in association with leukemia [18], the role of PP2A in neurodevelopment, related to proliferative effects in cell cycle dynamics, has been lately underlined [19]. Therefore, both considering phenotypic features related to *SETBP1-*HD and *SETBP1* function, this condition could be suggestive of as a RASopathy-like condition. Recently, other disorders caused by LoF pathogenic variants in other genes involved in the downstream regulation of the RAS/MAPK signaling have been as well related with clinical features partially overlapping with a RASopathy [20, 21]. Although fascinating, this hypothesis currently remains merely speculative, and is not confirmed in subject ID2 who does not present with clinical features particularly evocative of a RASopathy.

When considering the neurological and developmental presentation of *SETBP1*-HD, muscular hypotonia, DD, ID, and CAS-associated language impairment have been reported.

CAS is a severe neurodevelopmental disorder characterized by errors in speech sound production (usually inconsistent) and prosody. The involvement of neural planning, with consequent impairment of spatiotemporal parameters of movement sequences are at the basis of CAS [22].

CAS seems to occur in monogenic form [23]. Besides *FOXP2*, *BCL11A,* and *ERC1*, other six genes, among which *SETBP1*, have been recently studied in association with CAS, since they are expressed in crucial molecular pathways involved in the development of cerebral areas responsible of fluent spoken language acquisition. In particular, *SETBP1* is enriched in numerous brain areas involved in language development [24].

Subjects ID1 and ID3 displayed neuropsychiatric features already described in *SETBP1*-HD individuals, especially hypotonia, infantile febrile seizures, slowing at the EEG in absence of epileptiform activity, CAS, DD/ID, and ADHD. Subject ID2 seems to present a milder phenotype, mainly characterized by hypotonia, DD, language impairment, and hyperactivity.

We could hypothesize that other genomic imbalances in addition to *SETBP1* haploinsufficiency contribute to the more complex phenotype expressed by subjects ID1 and ID3.

In particular, subject ID1 also presented with a de novo 276 kb microdeletion at Xp22.1, including *DDX53*. This gene has recently emerged as a significant ASD risk factor, being involved in synaptic function [25, 26]; however, an overlapping genetic basis among ASD and ADHD has been evidenced [27], therefore this gene could contribute to our patient’s neurodevelopmental disorder, even if it does not seem to be fully causative by itself, especially considering his dysmorphic features and CAS, which are more typical of *SETBP1*-HD.

Subject ID3 presented with a more complex genomic situation: beyond the *SETBP1* haploinsufficiency, the patient also harbors large microdeletions, globally involving 36 OMIM genes, 13 out of them reporting as Disease Causing (Table 2). His clinical and facial features widely overlap with typical *SETBP1*-HD presentation, although we cannot exclude that the haploinsufficiency of some of the genes involved in the rearrangements may also have a contributing role in the patient’s phenotype and neurodevelopmental disorder.

**Table 2:** Subject ID 3: OMIM Disease Causing genes included within the 18q12.3q21.1 deleted region

Gene	Position	Disease	MIM number	Inheritance
*MLPH*	2q37.3	GRISCELLI SYNDROME	609,227	Autosomal recessive
*PER2*	2q37.3	ADVANCED SLEEP PHASE SYNDROME, FAMILIAL, 1	604,348	Autosomal dominant
*TRAF3IP1*	2q37.3	SENIOR-LOKEN SYNDROME 9	616,629	Autosomal recessive
*PDE10A*	6q27	DYSKINESIA, LIMB AND OROFACIAL, INFANTILE-ONSET	616,921	Autosomal recessive
		STRIATAL DEGENERATION (missense variants)	616,922	Autosomal dominant
*TBXT*	6q27	SACRAL AGENESIS WITH VERTEBRAL ANOMALIES	615,709	Autosomal recessive
		NEURAL TUBE DEFECTS, SUSCEPTIBILITY TO	182,940	Autosomal dominant
*MPC1*	6q27	MITOCHONDRIAL PYRUVATE CARRIER DEFICIENCY	614,741	Autosomal recessive
*SETBP1*	18q12.3	INTELLECTUAL DEVELOPMENTAL DISORDER, AUTOSOMAL DOMINANT 29	616,078	Autosomal dominant
		SCHINZEL-GIEDION MIDFACE RETRACTION SYNDROME (missense variants)	269,150	Autosomal dominant
*SLC14A1*	18q12.3	BLOOD GROUP, KIDD SYSTEM	111,000	
*EPG5*	18q12.3	VICI SYNDROME	242,840	Autosomal recessive
*ATP5F1A*	18q21.1	COMBINED OXIDATIVE PHOSPHORYLATION DEFICIENCY 22	616,045	Autosomal recessive
		MITOCHONDRIAL COMPLEX V (ATP SYNTHASE) DEFICIENCY, NUCLEAR TYPE 4B	615,228	Autosomal recessive
		MITOCHONDRIAL COMPLEX V (ATP SYNTHASE) DEFICIENCY (missense variants)	620,358	Autosomal dominant
*LOXHD1*	18q21.1	DEAFNESS	613,079	Autosomal recessive
*IER3IP1*	18q21.1	MICROCEPHALY, EPILEPSY, AND DIABETES SYNDROME 1	614,231	Autosomal recessive
*SMAD2*	18q21.1	CONGENITAL HEART DEFECTS, MULTIPLE TYPES, 8, WITH OR WITHOUT HETEROTAXY	619,657	Autosomal dominant
		LOEYS-DIETZ SYNDROME 6	619,656	Autosomal dominant

Subjects ID1 and ID2 present with a balanced reciprocal translocation involving chromosome 18 (at *SETBP1* locus) and an autosomal partner (chromosome 15 and 16 respectively).

Balanced chromosomal translocations (BCTs) are chromosomal material exchange without gain or loss of genetic material. However, it cannot be excluded that BCTs breakpoints interrupt disease genes, as occurring in about 6% of de novo BCTs [6]. In particular, it has been observed that neurodevelopmental disorders are quite frequent in patients with de novo BCTs implying gene disruption [15]. Translocations can occur as a result of a non-allelic homologous recombination (NAHR) process, usually mediated by low-copy repeats [28], or as a consequence of a double-strand breaks (DSBs) repair process. Non-homologous end-joining (NHEJ) is one of the major mechanisms used by eukaryotic cells for solving both physiological and pathological DSBs: the two broken DNA ends are recognized, modified for a compatible alignment restoration, and finally ligated. The modification step makes the process recognizable by the presence of sequence anomalies at the rejoining site, including a few nucleotides cleavage or addition [29]. The presence of repetitive elements (i.e. LTR, LINE or Alu sequences) or specific sequence motives promoting a DNA curvature, enhances NHEJ events.

The translocations detected in our subjects ID1 and ID2 were finely characterized by OGM as t(15;18)(q26.1;q12.3) and t(16;18)(p13.2;q12.3) and the breakpoints on chromosome 18 fell within *SETBP1* exon 6 and intron 2 respectively. Sequence analysis revealed in both cases the presence of additional unknown-origin material at the breakpoints (10 and 12 nucleotides respectively), and cryptic deletions (ranging from 4 bp to 6.4 kb), suggesting NHEJ as a possible mechanism.

Another male patient with a de novo BCT involving chromosome 18, t(12;18) (q22;q12.3), has been described so far: array-CGH excluded cryptic genomic imbalances, while whole genome sequencing revealed *SETBP1* disruption at intron 2 [16].

Subject ID3 harbored a complex genomic rearrangement, with multiple breakpoints on chromosome 2, 6, and 18. The rearrangement was not balanced and the deletions associated with some of the breakpoints had been previously detected by array-CGH, without any further clue for suspecting structural anomalies. The involved chromosome portions are too small in size to be detected by cytogenetics analysis (ranging from 3.9 kb to 1.4 Mb) and the real chromosomal architecture had been disclosed only by using OGM supported by WGS. In particular, 9 breakpoints have been detected on chromosome 18, within the region 18q12.3q21.1, 5 on chromosome 2 within the region 2q37.1q37.3, and 4 on chromosome 6, within the region 6q27. As well as for translocations, complex genomic rearrangements are usually the consequence of an erroneous microhomology-mediated recombination process [30]. A localized and chaotic shattering and reshuffling affecting one or few chromosomes is usually referred to as chromoanagenesis [31]. It consists in intra and inter-chromosomal rearrangements, with multiple breakpoints and possible associated deletions, arising from a one-step catastrophic event. The multiple genomic ruptures result in free chromosomal segments, which may reshuffle through an NHEJ mechanism in random orientation and position, or may eventually be lost, resulting in deletions. Subject ID3 is the product of a heterologous pregnancy due to sperm donation. The parents refused to be molecularly characterized.

We can’t exclude a de novo event due to chromotripsis caused by manipulative techniques during artificial reproductive techniques (ART) and sperm preparation, or a male germline mosaicism in the donor. Male germline mosaicism has been described as a possible cause of apparently de novo events of either pathogenic single variants or Copy Number Variations in individuals born after ART [32–34].

Also n Subject ID3, the breakpoint involving *SETBP1* falls in intron 2, as already reported in subject ID2 and in a patient by Vrkić Boban [16], suggesting this intron as a possible recombination hotspot. The previously detected 18q12.3q21.1 deletion, partially involving *SETBP1*, was shown to be associated with one of the structural events (the first BP on derivative 18). Several cases of deletions have been reported so far in scientific literature, at the point that deletions are considered one of the underlying molecular mechanisms of *SETBP1*-HD. However, no data are currently available about the genomic architecture, so that it is not possible to determine in how many of these cases the deletion represents the quantitative visible consequence of a more complex genomic rearrangement.

## Conclusions

This study reports on three patients with *SETBP1* haploinsufficiency, caused by chromosomal structural events interrupting the coding sequence of this gene. In two of them, the rearrangement was apparently balanced, while in the third one it was associated with deletions at some of the breakpoints, one of them partially including *SETBP1*. Molecular diagnosis was achieved through a combined approach based on OGM and WGS which allowed characterizing the translocations breakpoints, showing *SETBP1* disruption. The frequency of structural variants involving *SETBP1* is reasonably underestimated due to their challenging detection and characterization using standard techniques. Indeed, a definite diagnosis may be missed, in case of balanced rearrangements, overlooked by standard techniques (CMA and ES), or partially misunderstood, in case of unbalanced rearrangements presenting with a partial *SETBP1* deletion as the only detectable clue. The clinical revision of the *SETBP1*-HD presentation in our series is widely overlapping with those previously described.

Other studies are needed to confirm the implication of *SETBP1* in RAS pathway and to determine whether structural rearrangements can be considered a recurrent mechanism for SETBP1-HD, driven by a particular structure of the involved genomic region.

## Methods

### Chromosomal microarray analysis

DNA was isolated from peripheral blood by means of a QIAsymphony automatic extractor (QIAGEN, www.qiagen.com). Array-CGH analysis was performed by using an Agilent 4 × 180 K oligo-array platform, according to manufacturer’s instructions (www.agilent.com). Images were obtained by an Agilent DNA Microarray Scanner and analyses were performed by Agilent CytoGenomics software (v 5.3.0.14).

### Optical genome mapping (OGM) and structural variant calling

Optical genome mapping is a new non-sequencing imaging tool, providing high resolution information about the presence of copy number and structural variants all over the genome. The technology is based on the isolation of ultra-high molecular weight DNA, which are uniquely labelled, directly imaged and used for building an accurate physical genome map. Comparative analysis of the label patterns over long contiguous reads across the whole genome reveals the occurrence of both copy number variants and structural variants.

A fresh blood aliquot from the patients, collected in EDTA, was stored at -80 °C just after sampling. Ultra-high molecular weight (UHMW) DNA was extracted according to the manufacturer’s instructions (SP Frozen Human Blood DNA Isolation Protocol, Bionano Genomics), and enzymatically labeled by the DLE-1 Enzyme (Bionano Prep Direct Label and Stain Protocol). Labeled DNA was loaded on Saphyr chip and scanned on the Saphyr instrument (Bionano genomics, San Diego USA). Saphyr chip were ran to reach a minimum yield of 320 Gbp corresponding to 100X effective coverage. The de novo assembly and Variant Annotation Pipeline were executed on Bionano Solve software V3.6 using Human Genome Reference Consortium GRCh38 assembly as a reference for structural variants detection. Reporting and direct visualization of structural variants was done on Bionano Access V1.6.

### Whole-genome sequencing (WGS)

WGS was performed on genomic DNA in order to provide a fine characterization of the translocation events. Library preparation was carried out according to the manufacturer's protocol from DNA PCR-Free Library Prep (Illumina), and sequenced on a NovaSeq6000 (Illumina) platform. The obtained NGS (Next Generation Sequencing) assay presented a mean coverage of 35x, with Q30 bases around 87%. The TruSight Software Suite (Illumina) and the integrated DRAGEN platform and IGV software were used for alignment, variant calling and breakpoint data visualization. Sequencing data were aligned to the hg38 human reference genome.

### FISH analysis

FISH hybridization was performed on metaphases from peripheral blood of the two patients. Two custom locus-specific probes were designed on *SETBP1*, overlapping the 3’ and 5’ gene end respectively and labeled by different fluorochromes (SureDesign Agilent e-array https://earray.chem.agilent.com/suredesign/).

FISH slides were analyzed with Eclipse 80i (Nikon Instruments Europe B.V.), and images were captured using Genikon software (Nikon Instruments S.p.a.).

## Data Availability

The data that support the findings of this study are available upon request from the corresponding author. The data are not publicly available due to privacy or ethical restrictions.

## Abbreviations

AAC: augmentative and alternative communication
ADHD: Deficit hyperactivity disorder
ART: Artificial reproductive techniques
ASD: Autism spectrum disorder
BCT: Balanced chromosomal translocation
BP: Breakpoints
CAS: Childhood apraxia of speech
CMA: Chromosomal microarray analysis
CSR: Chromosomal structural rearrangements
DD: Developmental delay
ES: Exome sequencing
FISH: Fluorescence in situ hybridization
ID: Intellectual disability
LoF: loss-of-function
MRD29: Intellectual disability, autosomal dominant 29
MRI: Magnetic resonance imaging
MS-MLPA: Methylation specific-Multiplex Ligation-dependent Probe Amplification
NAHR: Non-allelic homologous recombination
NGS: Next generation sequencing
NHEJ: Non-homologous end-joining
NS: Noonan syndrome
OFC: Occipito-frontal circumference
OGM: Optical genome mapping
SD: Standard deviation
*SETBP1*: SET binding protein 1
*SETBP1*-HD: *SETBP1* haploinsufficiency disorder
WGS: Whole genome sequencing

## References

[1] Greenstein RM, Reardon MP, Chan TS, Middleton AB, Mulivor RA, Greene AE, Coriell LL (1980). An (X;11) translocation in a girl with Duchenne muscular dystrophy. Repository identification No. GM1695. Cytogenet Cell Genet..

[2] Tommerup N (1993). Mendelian cytogenetics. Chromosome rearrangements associated with mendelian disorders. J Med Genet..

[3] Bache I, Hjorth M, Bugge M, Holstebroe S, Hilden J, Schmidt L, Brondum-Nielsen K, Bruun-Petersen G, Jensen PK, Lundsteen C, Niebuhr E, Rasmussen K, Tommerup N (2006). Systematic re-examination of carriers of balanced reciprocal translocations: a strategy to search for candidate regions for common and complex diseases. Eur J Hum Genet.

[4] Higgins AW, Alkuraya FS, Bosco AF, Brown KK, Bruns GA, Donovan DJ, Eisenman R, Fan Y, Farra CG, Ferguson HL, Gusella JF, Harris DJ, Herrick SR, Kelly C, Kim HG, Kishikawa S, Korf BR, Kulkarni S, Lally E, Leach NT, Lemyre E, Lewis J, Ligon AH, Lu W, Maas RL, MacDonald ME, Moore SD, Peters RE, Quade BJ, Quintero-Rivera F, Saadi I, Shen Y, Shendure J, Williamson RE, Morton CC (2008). Characterization of apparently balanced chromosomal rearrangements from the developmental genome anatomy project. Am J Hum Genet.

[5] Jacobs PA, Browne C, Gregson N, Joyce C, White H (1992). Estimates of the frequency of chromosome abnormalities detectable in unselected newborns using moderate levels of banding. J Med Genet.

[6] De WD (1991). novo balanced chromosome rearrangements and extra marker chromosomes identified at prenatal diagnosis: clinical significance and distribution of breakpoints. Am J Hum Genet.

[7] Buysse K, Menten B, Oostra A, Tavernier S, Mortier GR, Speleman F (2008). Delineation of a critical region on chromosome 18 for the del(18)(q122q211) syndrome. Am J Med Genet Part A..

[8] Cody JD, Sebold C, Malik A, Heard P, Carter E, Crandall A (2007). Recurrent interstitial deletions of proximal 18q: a new syndrome involving expressive speech delay. Am J Med Genet Part A.

[9] Filges I, Shimojima K, Okamoto N, Röthlisberger B, Weber P, Huber AR (2011). Reduced expression by SETBP1 haploinsufficiency causes developmental and expressive language delay indicating a phenotype distinct from Schinzel-Giedion syndrome. J Med Genet.

[10] Jansen NA, Braden RO, Srivastava S, Otness EF, Lesca G, Rossi M (2021). Clinical delineation of SETBP1 haploinsufficiency disorder. Eur J Hum Genet.

[11] Leonardi E, Bettella E, Pelizza MF, Aspromonte MC, Polli R, Boniver C, Sartori S, Milani D, Murgia A (2020). Identification of SETBP1 mutations by gene panel sequencing in individuals with intellectual disability or with "developmental and epileptic encephalopathy". Front Neurol..

[12] Wang H, Gao Y, Qin L, Zhang M, Shi W, Feng Z, Guo L, Zhu B, Liao S (2023). Identification of a novel de novo mutation of SETBP1 and new findings of SETBP1 in tumorgenesis. Orphanet J Rare Dis.

[13] Morgan A, Srivastava S, Duis J, et al. SETBP1 Haploinsufficiency Disorder. 2021 Nov 18. In: Adam MP, Mirzaa GM, Pagon RA, et al., editors. GeneReviews® [Internet]. Seattle (WA): University of Washington, Seattle; 1993–2023. https://www.ncbi.nlm.nih.gov/books/NBK575336/. 34807554

[14] Dremsek P, Schwarz T, Weil B, Malashka A, Laccone F, Neesen J (2021). Optical genome mapping in routine human genetic diagnostics-its advantages and limitations. Genes (Basel).

[15] Redin C, Brand H, Collins RL (2017). The genomic landscape of balanced cytogenetic abnormalities associated with human congenital anomalies. Nat Genet.

[16] Vrkić Boban I, Sekiguchi F, Lozić M, Miyake N, Matsumoto N, Lozić B (2020). A novel *SETBP1* gene disruption by a de novo balanced translocation in a patient with speech impairment, intellectual, and behavioral disorder. J Pediatr Genet.

[17] Morgan A, Braden R, Wong MMK, Colin E, Amor D, Liégeois F (2021). Speech and language deficits are central to SETBP1 haploinsufficiency disorder. Eur J Hum Genet..

[18] Carratt SA, Braun TP, Coblentz C, Schonrock Z, Callahan R, Curtiss BM (2021). Mutant SETBP1 enhances NRAS-driven MAPK pathway activation to promote aggressive leukemia. Leukemia.

[19] Antonyan L, Ernst C (2022). Putative roles of SETBP1 dosage on the SET oncogene to affect brain development. Front Neurosci.

[20] Kent OA, Saha M, Coyaud E, Burston HE, Law N, Dadson K, Chen S, Laurent EM, St-Germain J, Sun RX, Matsumoto Y, Cowen J, Montgomery-Song A, Brown KR, Ishak C, Rose J, De Carvalho DD, He HH, Raught B, Billia F, Kannu P, Rottapel R (2020). Haploinsufficiency of RREB1 causes a Noonan-like RASopathy via epigenetic reprogramming of RAS-MAPK pathway genes. Nat Commun.

[21] Priolo M, Zara E, Radio FC, Ciolfi A, Spadaro F, Bellacchio E, Mancini C, Pantaleoni F, Cordeddu V, Chiriatti L, Niceta M, Africa E, Mammì C, Melis D, Coppola S, Tartaglia M (2023). Clinical profiling of MRD48 and functional characterization of two novel pathogenic RAC1 variants. Eur J Hum Genet.

[22] Terband H, Namasivayam A, Maas E, van Brenk F, Mailend ML, Diepeveen S (2019). Assessment of childhood apraxia of speech: a review/tutorial of objective measurement techniques. J Speech Lang Hear Res.

[23] Graham SA, Fisher SE (2015). Understanding language from a genomic perspective. Annu Rev Genet.

[24] Eising E, Carrion-Castillo A, Vino A, Strand EA, Jakielski KJ, Scerri TS (2019). A set of regulatory genes co-expressed in embryonic human brain is implicated in disrupted speech development. Mol Psychiatry.

[25] Ross PJ, Zhang WB, Mok RSF, Zaslavsky K, Deneault E, D'Abate L (2020). Synaptic dysfunction in human neurons with autism-associated deletions in PTCHD1-AS. Biol Psychiatry.

[26] Pinto D, Pagnamenta AT, Klei L, Anney R, Merico D, Regan R (2010). Functional impact of global rare copy number variation in autism spectrum disorders. Nature.

[27] Ronald A, Simonoff E, Kuntsi J, Asherson P, Plomin R (2008). Evidence for overlapping genetic influences on autistic and ADHD behaviours in a community twin sample. J Child Psychol Psychiatry.

[28] Gu W, Zhang F, Lupski JR (2008). Mechanisms for human genomic rearrangements. Pathogenetics.

[29] Symington LS, Gautier J (2011). Double-strand break end resection and repair pathway choice. Annu Rev Genet.

[30] Zhang F, Carvalho CM, Lupski JR (2009). Complex human chromosomal and genomic rearrangements. Trends Genet.

[31] Pellestor F, Gatinois V (2020). Chromoanagenesis: a piece of the macroevolution scenario. Mol Cytogenet.

[32] Chalas C, Receveur A, Frydman N, Massin N, Tachdjian G, Drouineaud V, Benachi A, Patrat C, Petit FM (2020). A case of germline mosaicism for a 7q32.1q33 deletion in a sperm donor: consequences on pregnancy follow-up and recommendations. Basic Clin Androl..

[33] Ejerskov C, Farholt S, Skovby F, Vestergaard EM, Haagerup A (2016). Clinical presentations of 23 half-siblings from a mosaic neurofibromatosis type 1 sperm donor. Clin Genet.

[34] Callum P, Messiaen LM, Bower PV, Skovby F, Iger J, Timshel S (2012). Gonosomal mosaicism for an NF1 deletion in a sperm donor: evidence of the need for coordinated, long-term communication of health information among relevant parties. Hum Reprod.

